# Evaluating Apple Inc Mobility Trend Data Related to the COVID-19 Outbreak in Japan: Statistical Analysis

**DOI:** 10.2196/20335

**Published:** 2021-02-15

**Authors:** Junko Kurita, Yoshiyuki Sugishita, Tamie Sugawara, Yasushi Ohkusa

**Affiliations:** 1 Department of Nursing Tokiwa University Mito, Ibraki Japan; 2 National Institute of Infectious Diseases Tokyo Japan

**Keywords:** peak, COVID-19, effective reproduction number, mobility trend data, Apple, countermeasure

## Abstract

**Background:**

In Japan, as a countermeasure against the COVID-19 outbreak, both the national and local governments issued voluntary restrictions against going out from residences at the end of March 2020 in preference to the lockdowns instituted in European and North American countries. The effect of such measures can be studied with mobility data, such as data which is generated by counting the number of requests made to Apple Maps for directions in select countries/regions, sub-regions, and cities.

**Objective:**

We investigate the associations of mobility data provided by Apple Inc and an estimate an an effective reproduction number R(*t*).

**Methods:**

We regressed R(*t*) on a polynomial function of daily Apple data, estimated using the whole period, and analyzed subperiods delimited by March 10, 2020.

**Results:**

In the estimation results, R(*t*) was 1.72 when voluntary restrictions against going out ceased and mobility reverted to a normal level. However, the critical level of reducing R(*t*) to <1 was obtained at 89.3% of normal mobility.

**Conclusions:**

We demonstrated that Apple mobility data are useful for short-term prediction of R(*t*). The results indicate that the number of trips should decrease by 10% until herd immunity is achieved and that higher voluntary restrictions against going out might not be necessary for avoiding a re-emergence of the outbreak.

## Introduction

The rapid spread of COVID-19, the disease caused by SARS-CoV-2, continues to have a substantial impact. At the end of October 2020, the World Health Organization reported approximately 46 million COVID-19 cases and more than one million fatalities. Japan, however, reported only approximately 100,000 cases with 1700 deaths, and at 769.2 per one million residents, the country’s incidence rate was considerably lower than the global average of 5974 per million. Japan’s case fertility rate, however, was comparable with the worldwide rate (1.7% vs 2.2%); that is, the country showed fewer patients but a moderate case fertility rate. Understanding the dynamics of Japan’s COVID-19 outbreak has been important for planning and evaluating countermeasures.

As a countermeasure against the COVID-19 outbreak in Japan, both the national and local governments issued voluntary restrictions against going out at the end of March 2020 in preference to lockdowns such as those instituted in European and North American countries [1]. However, it is unclear exactly how voluntary restriction against going out requirements affected the outbreak compared with the country’s post–voluntary restriction against going out statistics. The outbreak in Japan reached its first peak at the beginning of April 2020, but it was unclear why the peak occurred at that time. Thus, understanding the associations between the voluntary restriction against going out implementation and the first peak is urgent for controlling subsequent waves in Japan.

Susceptible-infected-recovered (SIR) models for COVID-19 incorporating countermeasures have emphasized the date that countermeasures were initiated [2,3]. However, at least in Japan, voluntary restrictions against going out expanded gradually; therefore, all-or-nothing approaches such as SIR models might not be appropriate. A more continuous variable is expected to be necessary to represent voluntary restriction against going out compliance over time.

Several companies, including Apple Inc and Alphabet Inc (hereinafter Apple and Google, respectively) worldwide, and Nippon Telegraph and Telephone (NTT) and East and West Japan Railway (JR) companies in Japan, have made available relevant data on such variables. Apple, the front-runner of this service, began providing data from January 13, 2020, specifically the daily ratio of the number of trips from homes to activities by transportation type (driving, transit, and walking) [4]. This data is generated by counting the number of requests made to Apple Maps for directions in select countries/regions, sub-regions, and cities. For privcy protection, data that is sent from users’ devices to the Apple Maps service is associated with random, rotating identifiers; thus Apple claims to not have a profile of the movements and searches of individual users. Using trip information provided by Apple, we examined various associations and estimated the effective reproduction number R(*t*).

## Methods

Applying a simple SIR model [2,3,5] to the epidemic curve in Japan, with its 120 million population, we assumed an incubation period that conformed to the empirical distribution in Japan; we used the number of patients who were symptomatic reported by the Ministry of Labour, Health and Welfare (MLHW) for February 10 to April 30, 2020, published on May 13, 2020 [6]. We excluded some patients from the data: persons presumed to have been infected abroad or as passengers on the Diamond Princess (the Ministry presumed that these patients did not represent a community-acquired infection in Japan). For some patients who were symptomatic, their onset dates were unknown; for these patients, we estimated the dates from an empirical distribution with duration extending from the onset to the report date among patients for whom the onset date had been reported.

In detail, we estimated the unknown onset dates as follows. Letting *f*(*k*) represent this empirical distribution and *N_t_* denote the number of patients for whom onset dates were not available published at date *t*, the number of patients for whom the onset date was known is *t* − 1. The number of patients for whom onset dates were not available was estimated as *f*(1)*N_t_*. Similarly, the number of patients with onset date *t* − 2 for whom onset dates were not available was estimated as *f*(2)*N_t_*. Hence, we estimated the total number of patients for whom the onset date was not available, considering an onset date of *s*, as Σ*_k _*_= 1_*f*(*k*)*N_s_* + *k* for the long duration extending from *s*.

Moreover, reporting delays were possible for the MLHW’s published data; that is, if *s* + *k* was larger than that in the current period *t*, then *s *+ *k* represented the future for period *t*, and for this reason, *Ns *+ *k* was not observable. This reporting delay engendered the underestimation of the number of patients, and thus, it had to be adjusted as *Σ_k = _*_1_*^t − s^f(k)N_s_ + k/Σ_k = _*_1_*^t − s^f*(*k*). Similarly, we expected that patients for whom the onset dates were available would be affected by the reporting delay, leading to *M_s_*|*_t_*/Σ*_k _*_= 1_*^t − s^f*(*k*), where *M_s_*|*t* represents the reported number of patients for whom onset dates were within period *s*, extending until current period *t*.

We defined R(*t*) as the number of patients who were infected on day *t* divided by the number of patients who were presumed to be infectious. We calculated the number of patients who were infected from the epidemic curve by the onset date using a distribution of the incubation period. The distribution of infectiousness in symptomatic and asymptomatic cases was assumed to be 30% on the onset day, 20% on the following day, and 10% for the subsequent 5 days [7].

For analyses, we used Apple’s average daily information on the three types of trips as well as their ratios using a root search function on Apple Maps on each day compared with that on January 13, 2020. Apple provides no information regarding how many users or proportions of Apple users used this function.

To elucidate the associations between R(*t*) and Apple data, we regressed R(*t*) on a polynomial function of daily data. We determined the order of the polynomial function stepwise from the model including only a linear term to the model, including a higher order if all estimated coefficients were significant. Besides the estimation using the whole period, we analyzed subperiods before and after March 10, 2020.

**Figure 1:**
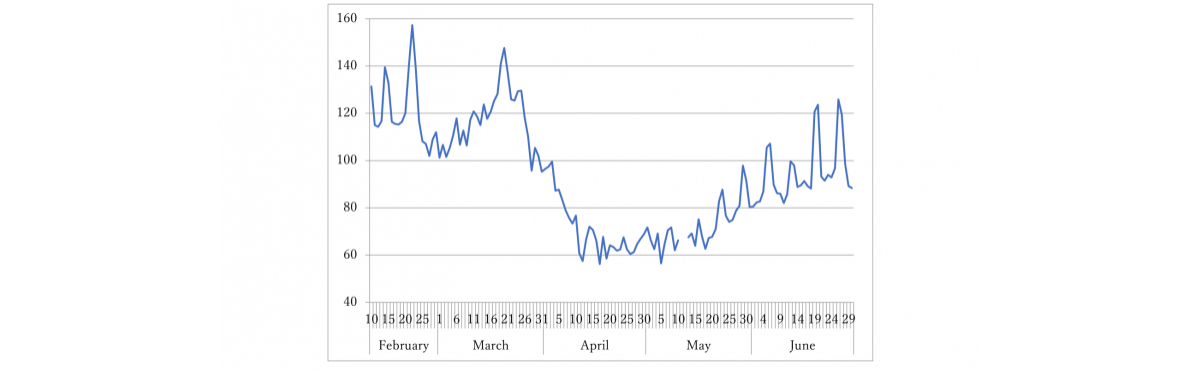
Apple data in Japan from February 10 to the end of June 2020. The line represents the ratio of the number of trips from homes to activities by transportation type (driving, transit, and walking) on January 13, 2020. Apple data for May 12 and 13, 2020, were missing. The prediction in the period was also missing.

To intuitively understand the estimation result, we predicted R(*t*) when voluntary restriction against going out ceased and Apple data reached 100. We also sought the highest value of Apple data that implied R(*t*)<1 by a grid search from 100. To confirm the predictive power of the model, we predicted R(*t*) based on available Apple data on day *t* prospectively for the subsequent 2 months until the end of June 2020 (Figure 1); then, we compared the result with the observed R(*t*) on day *s*>*t* + 40 to avoid uncertainty because of reporting delays. We inferred significance at the 5% level.

## Results

**Figure 2:**
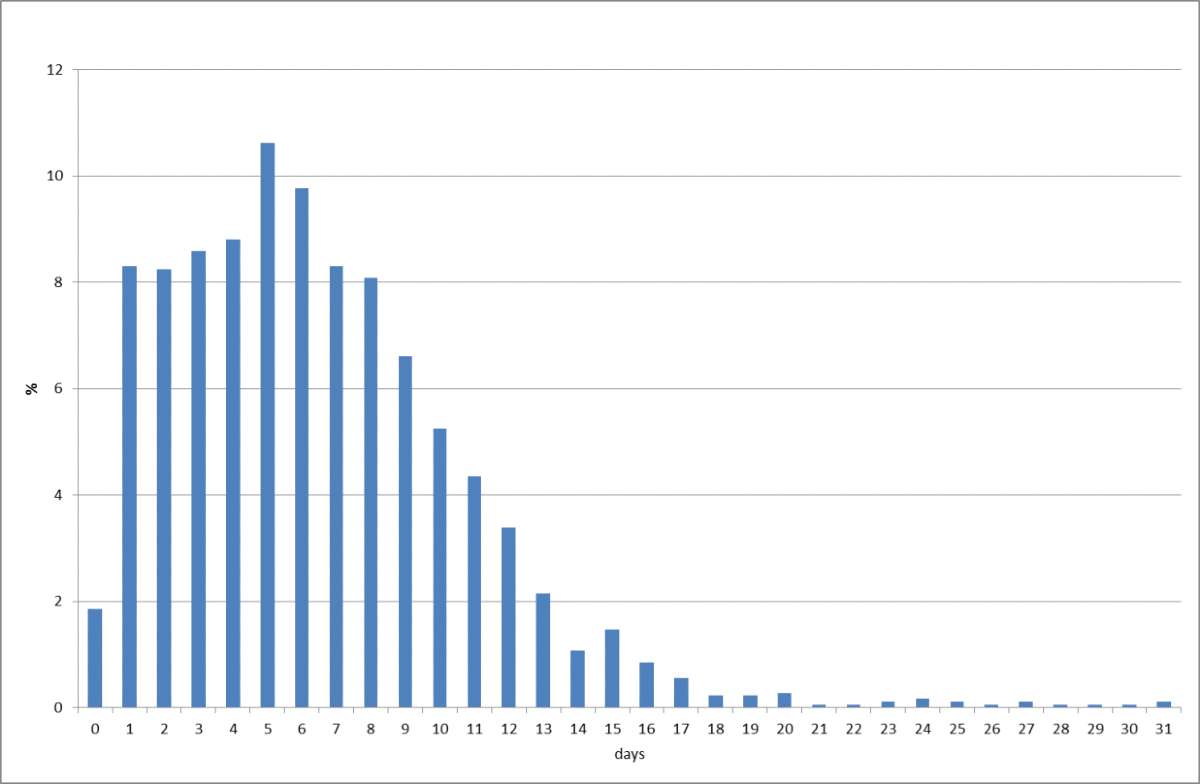
Empirical distribution of duration from onset to report by Ministry of Labour, Health and Welfare in Japan. Bars represent the probability of duration from onset to report based on 657 patients for whom the onset date was available.

**Figure 3:**
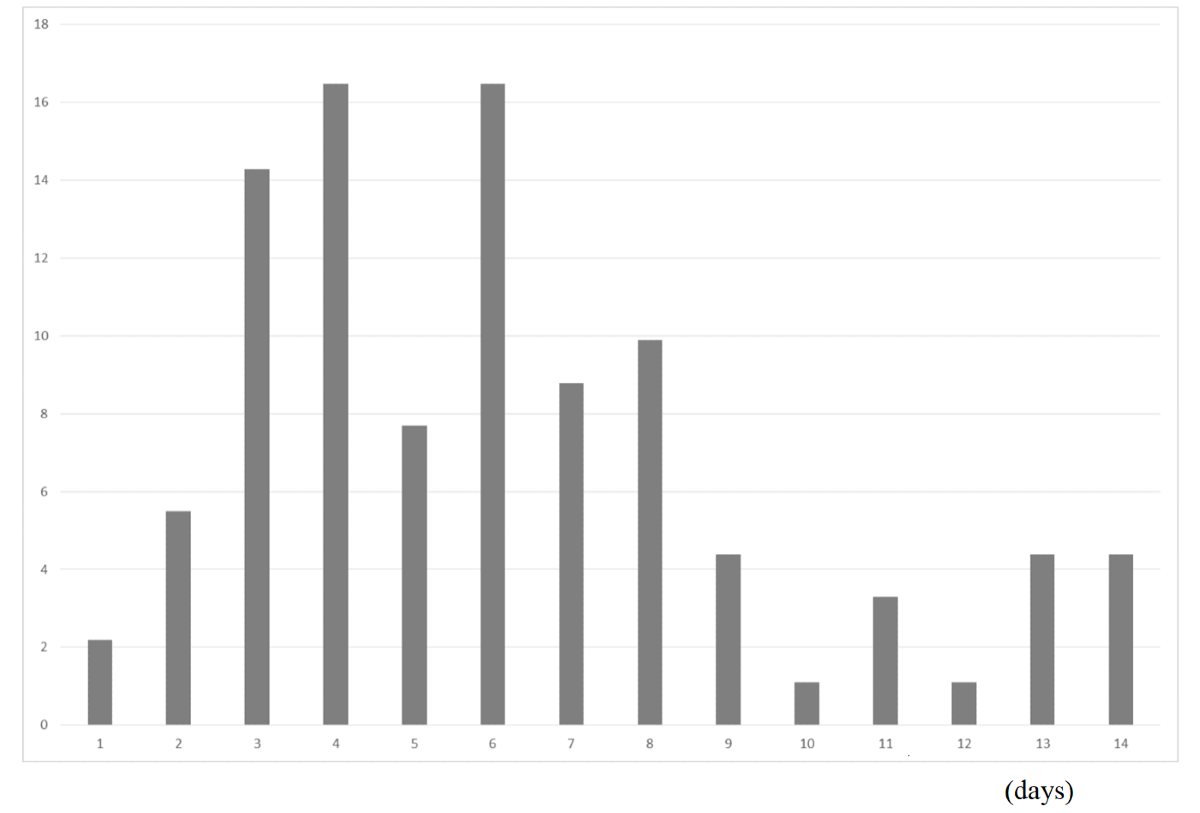
Empirical distribution of the incubation period published by Ministry of Labour, Health and Welfare (MLHW) in Japan. Bars show the distribution of incubation periods for 91 cases for which MLHW Japan had published the exposure date and the onset date. The patients for whom incubation was longer than 14 days are included in the bar shown for day 14.

From February 10 to April 30, 2020, in Japan, 13,967 community-acquired cases were identified, excluding asymptomatic cases. Figure 2 presents an empirical distribution of the duration of onset to a report in Japan, showing a maximum delay of 30 days. Figure 3 depicts the empirical distribution of incubation periods among 91 cases for which the MLHW had published an exposed date and an onset date. The mode was 6 days, and the average was 6.6 days.

Table 1 presents the estimation result. During the whole period and after the March 10, 2020, period, we selected up to cubic terms; however, before the March 10 period, we selected the quadratic term. The estimated coefficients in the whole period and after the March 10 period were similar, although they differed greatly from the subperiod before March 10.

**Table 1:** Results of estimate R(*t*) for Apple data.^a^

Terms	Whole period (N=81)		Before March 10, 2020 (n=29)		After March 10, 2020 (n=52)	
	Estimate coefficient	*P* value	Estimate coefficient	*P* value	Estimate coefficient	*P* value
Linear term	−0.705	<.001	0.451	.004	−0.408	.008
Quadratic term	0.007	<.001	−0.002	.006	0.004	.008
Cubic term	−0.228*10−4	<.001	N/A^b^	N/A	−0.126*10−4	.02
Constant	21.55	<.001	−27.26	.005	13.51	.008
Adjusted *R*^2^	0.701	N/A	0.349	N/A	0.891	N/A

^a^The dependent variable was R(*t*), and the explanatory variables were the polynomial function of Apple data.

^b^N/A: not applicable.

**Figure 4:**
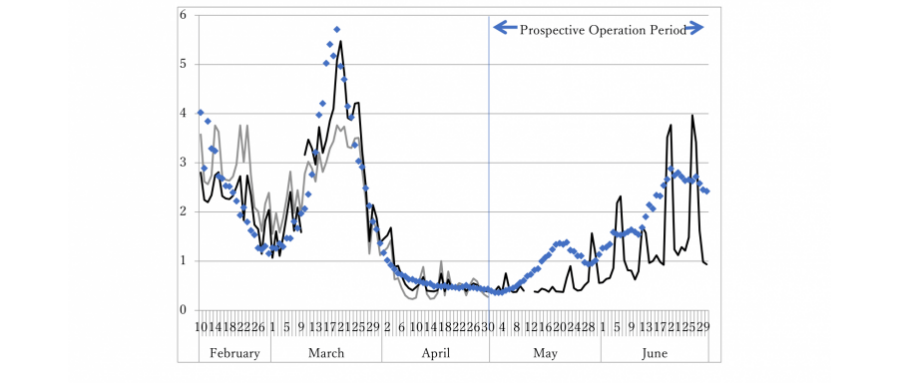
R(*t*) and prediction from Apple data. The two black lines represent the prediction from Apple data in the subperiods before and after March 10, 2020; the gray line represents the prediction in the whole period. Dots represent the observed R(*t*). Apple data on May 12 and 13 were missing, and the prediction in the period was also missing. Until the end of April 2020, the prediction from Apple data was retrospective; from the beginning of May 2020, the prediction was prospective, although observed R(*t*) was calculated as of the date on t + 40.

Figure 4 depicts the R(*t*) and the predicted line based on Apple data in the whole period and the two subperiods. The model clearly shows poor fit in the whole period, especially around the peak at the end of March 2020. Conversely, the prediction in the post–March 10 subperiod shows a clearly good fit around the peak.

The estimation result indicates that R(*t*) was 1.72 when voluntary restriction against going out ceased and mobility reverted to a normal level, which was 100 in the Apple data. Therefore, complete cessation of voluntary restrictions against going out would reinitiate the outbreak. We found in the Apple data that the critical level of R(*t*)<1 was 89.3.

Prospective operation using Apple data from the beginning of May 2020 for 2 months is shown on the right-hand side of Figure 3. The correlation coefficient for the actual observed R(*t*) and prospective prediction from Apple data for this period was 0.6433, which was significant.

## Discussion

### Principal Findings

Here, we demonstrated that Apple data are useful for short-term prediction of R(*t*). Figure 3 suggests that a model with two subperiods might be preferable to the whole model. Such a model might be consistent with the apparent change in the dominant virus among SARS-CoV-2 that occurred in March 2020 [8]. The estimations seem to indicate that the Wuhan-originated strain was dominant before the end of March but that a mutated virus subsequently became dominant in European countries and the United States. These two virus subtypes showed different associations with the mobility data and might have different infectiousness.

In the prospective operation period, the prediction using Apple data correlated significantly with the ultimately observed R(*t*), although the correlation among them was only slightly higher than the finding from the retrospective analysis until the end of April 2020; this finding is likely attributable to prospective operation. Furthermore, because the state of emergency declaration was canceled at the end of May 2020, the behavior of the population could be expected to have changed around then; for example, the intensity of meetings might have been lower or more mask wearing and longer distancing might be used for conversations. Under such circumstances, Apple data might lose their predictive power.

Current results obtained using Apple data indicate a critical level of R(*t*)<1 as 89.3. Consequently, a 10% reduction in trips from home, likely for more than 1 year, will be necessary until the so-called herd immunity is achieved; if this decrease is achieved, full voluntary restrictions against going out might not be necessary to avoid another outbreak. Current voluntary restriction against going out data indicate a 40% reduction from Apple data. Therefore, restrictions against excursions can be relaxed by 30 percentage points; such relaxation might include restarting schools or private events and sports that involve small numbers of players, little player contact, and large outside spaces, but large entertainment events and professional sports events with numerous participants might continue to be risky. In other words, it is possible to monitor a partial relaxation of voluntary restrictions against going out consistent with a controlled outbreak including monitoring economic activity to maintain R(*t*)<1 but not extremely low. Apple data might support fine control of the outbreak and acceptable levels of social interaction.

In an unpublished study using Google data [9], a 70% reduction in going out was necessary to control the outbreak in Spain, which is different from the degree required in Japan. That in Spain was seven times higher than that in Japan. In fact, the outbreak in Japan was controlled using voluntary restrictions against going out without lockdown, yet the country’s necessary degree of reduction in going out was seven times lower than Spain’s.

A COVID-19 advisory council in Japan has required an 80% reduction since the emergency declaration on April 8, 2020 [10], and trips outside the home have decreased 40% from normal levels. However, the outbreak has been decreasing; it has become apparent that the requirement was stricter than necessary, which has at least produced some evidence of what a request of 80% reduction is likely to elicit from citizens. Our study results indicate that a mere 11% reduction might be sufficient to control an outbreak. In the early stage of the outbreak, R_0_ of 0.6 was inferred [11], which indicated that an outbreak would never have occurred in Japan. Such estimates might also have led to misguided countermeasures in Japan that necessitated adherence to contact tracing for cluster detection.

Earlier researchers [12] who used worldwide Apple and Google data examined only linear terms, whereas results show that higher terms might be necessary for predicting R(*t*). As such, the higher predictive power from Apple data suggests that R(*t*) predicted using Apple data might be more reliable.

Particularly, the estimated R(*t*) over a few days was less precise because the virus incubation period is approximately 6 days on average, and increasing R(*t*) over time up to 6 days was a widely observed phenomenon. Conversely, Apple data are available the next day, and thus, the latest R(*t*) from Apple data might be predicted.

We used Apple data exclusively, rather than data from Google or other sites, because Apple began providing the earliest data, and it has also continued to provide data on a timely basis with only 2 days of delay. Google data are delayed longer than the Apple data, and although the NTT and JR are likely delayed by only 1 day or half a day, they began compiling their data later. Additionally, NTT and JR data were not published systematically or were not made available for public use. Therefore, Apple’s data were most appropriate for evaluating the outbreak dynamics and predicting them prospectively.

This study has some limitations. First, R(*t*) was not determined for the number of contacts only. It depends on other circumstances such as the proportion of people who are susceptible and the infectiousness of people who are asymptomatic, and we did not incorporate these into our calculations. Considering such data would have required an SIR model that included asymptomatic cases, which we anticipate to be a challenge for future research.

Second, we examined this problem only for the entirety of Japan, but Apple also provides this information by prefecture; therefore, we could have extended our analyses to affected prefectures such as Tokyo, Osaka, and Hokkaido.

Third, it should be highlighted that Apple data show the proportions of users leaving their residences, but the data do not directly reflect numbers or rates of contacts; in other words, Apple data show no intensity of respective contacts. In fact, the measurements of contact intensity are difficult, but we consider meeting this challenge as a future research objective.

Fourth, although Apple data were better than those from other sources, Apple users might be limited to a particular population of young or healthy people, whereas information from NTT or JR might not be limited to those users. Thus, combining Apple data with data from these other sources might yield better results than those from this study, although these data have some disadvantages compared with the Apple data. Assessing and overcoming such limitations are challenges for future research efforts.

### Conclusions

We demonstrated that mobility data from Apple Inc are useful for short-term prediction of R(*t*). Specifically, we determined that reducing trips from home by 11% was sufficient to maintain R(*t*)<1. We should examine the Apple data carefully to evaluate the effects of countermeasures.

## Abbreviations

JR: Japan Railway
MLHW: Ministry of Labour, Health and Welfare
NTT: Nippon Telegraph and Telephone
SIR: susceptible-infected-recovered
